# A Novel Cascade Classifier for Automatic Microcalcification Detection

**DOI:** 10.1371/journal.pone.0143725

**Published:** 2015-12-02

**Authors:** Seung Yeon Shin, Soochahn Lee, Il Dong Yun, Ho Yub Jung, Yong Seok Heo, Sun Mi Kim, Kyoung Mu Lee

**Affiliations:** 1 Department of Electrical and Computer Engineering, ASRI, Seoul National University, Seoul, Republic of Korea; 2 Department of Electronic Engineering, Soonchunhyang University, Asan, Republic of Korea; 3 Division of Computer and Electronic Systems Engineering, Hankuk University of Foreign Studies, Yongin, Republic of Korea; 4 Department of Electrical and Computer Engineering, Ajou University, Suwon, Republic of Korea; 5 Department of Radiology, Seoul National University Bundang Hospital, Seongnam, Republic of Korea; Jiangnan University, CHINA

## Abstract

In this paper, we present a novel cascaded classification framework for automatic detection of individual and clusters of microcalcifications (*μ*C). Our framework comprises three classification stages: i) a random forest (RF) classifier for simple features capturing the second order local structure of individual *μ*Cs, where non-*μ*C pixels in the target mammogram are efficiently eliminated; ii) a more complex discriminative restricted Boltzmann machine (DRBM) classifier for *μ*C candidates determined in the RF stage, which automatically learns the detailed morphology of *μ*C appearances for improved discriminative power; and iii) a detector to detect clusters of *μ*Cs from the individual *μ*C detection results, using two different criteria. From the two-stage RF-DRBM classifier, we are able to distinguish *μ*Cs using explicitly computed features, as well as learn implicit features that are able to further discriminate between confusing cases. Experimental evaluation is conducted on the original Mammographic Image Analysis Society (MIAS) and mini-MIAS databases, as well as our own Seoul National University Bundang Hospital digital mammographic database. It is shown that the proposed method outperforms comparable methods in terms of receiver operating characteristic (ROC) and precision-recall curves for detection of individual *μ*Cs and free-response receiver operating characteristic (FROC) curve for detection of clustered *μ*Cs.

## Introduction

Breast cancer is the most common cancer in women worldwide and the second most common cancer overall [1]. In the US, nearly 1 in 36 (2.8%) women die of breast cancer related illnesses, and about 1 in 8 (12%) will develop invasive breast cancer during their lifetime [2]. The majority of cases of breast cancer are associated with clusters of microcalcifications (*μ*C), which are tiny calcium deposits that appear as little white spots on a mammogram [3]. Thus, identifying clusters of *μ*Cs is crucial for the detection of breast cancer. Thus, a significant amount of research on computer aided detection (CAD) of *μ*Cs in mammograms has been conducted.

**Fig 1:**
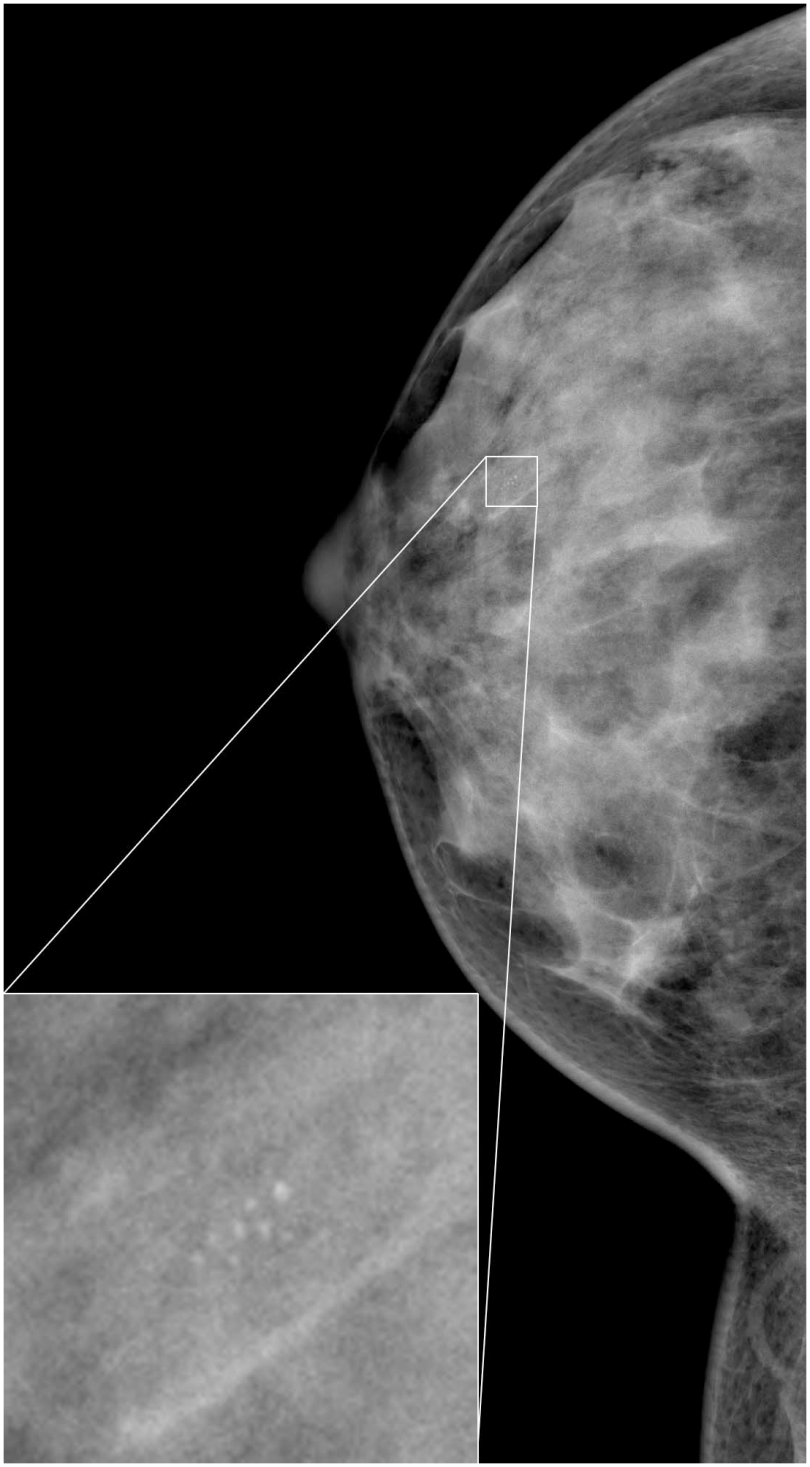
An example mammogram and the enlarged view of a contained microcalcification cluster. Intensity windowing is applied for improved visibility.

Fig 1 shows a digital mammogram containing a cluster of *μ*Cs. The detection of individual *μ*Cs is generally difficult due to their diversified morphologies, small size and surrounding tissues. Moreover, many false positives are likely to occur due to dense breast tissue, cysts, or noise with similar local appearance with *μ*Cs.

Previous works can be classified into two categories, *image processing based methods* and *learning based methods*. Many early works were based on image processing techniques, including image enhancement [4, 5], filtering [6–8], morphological operations [9], and coding [10–12]. For the most part, these methods are designed by experts with specific targets regarding the characteristic appearance that it aims to distinguish. Thus, while these techniques are successful for the targeted types of *μ*Cs, they may be limited for *μ*Cs with subtle local appearance variations, since it is extremely difficult to manually examine and consider all possible cases for a large amount of real data.

More recently, several learning based methods have been developed based on developments in discriminative local feature descriptors and machine learning methods for classification. During the past decade, methods using different machine learning methods such as boosting [13], support vector machines (SVM) [14] and relevance vector machine (RVM) [15] have been proposed. In these methods, selecting the appropriate feature is a critical issue for improving detection quality. The appropriate feature must effectively represent the discerning characteristics between the *μ*Cs and normal tissue. Usually, the feature selection procedure needs expert knowledge and requires time-consuming hand-tuning. For example, in the method by Oliver et al. [13], features extracted from a bank of filters are selectively used in a boosted classifier framework.

Previous works can also be classified by the specific configuration of *μ*Cs in which to detect. Malignant tissues often occur as closely clustered *μ*Cs within a limited region. Therefore, detection of clusters of *μ*Cs is of higher clinical importance than the detection of individual *μ*Cs.

Several works thus aim to directly detect *μ*C clusters. An example is the work by Papadopoulos et al. [16, 17], where a *μ*C cluster is detected by classifying features characterizing either an individual, or a group of, *μ*Cs in a particular region of interest (ROI).

Other works aim to detect individual *μ*Cs first, and then detect *μ*C clusters. The assumption here is that, compared to clusters of *μ*Cs, the appearance of individual *μ*Cs is more consistent and thus can be better modelled. And, based on robust detections of individual *μ*Cs, the *μ*C clusters can accurately be detected as well. In the work by El-Naqa et al. [14], a method comprising a pixel-wise SVM classifier trained to detect individual *μ*Cs and a successive enhancement learning (SEL) algorithm to enhance accuracy is proposed. In the work by Wei et al. [15], a linear classifier and a non-linear RVM classifier are combined in a two-stage network to enhance accuracy and efficiency. In both works, the evaluation of individual *μ*C detection is conducted not on actual clinical cases where only an extremely small number of pixels are *μ*Cs but on comparatively well-balanced test samples. In the work by Bria et al. [18], a ranking based cascade *μ*C detector based on classifiers for Haar-like features is followed by a classifier for *μ*C clusters. Their evaluations on custom datasets show competitive performance compared to high-end commercial systems.

In this paper, we present a new cascaded classification framework for automatic detection of *μ*Cs and *μ*C clusters. Our framework comprises three classification stages, where the first and second stages enable accurate and robust detection of individual *μ*Cs, while the final stage aggregates the individual *μ*Cs to detect *μ*C clusters. More specifically, a random forest (RF) classifier [19, 20] learned using simple features capturing the second order local structure of *μ*Cs is applied to efficiently eliminate non-*μ*C pixels in the target mammogram. *μ*C candidates determined in the first stage are then further classified using a more complex discriminative restricted Boltzmann machine (DRBM) classifier [21] to obtain individual *μ*C detections. By dividing the detection process into two separate and complementary classification stages, we are able to utilize implicit characteristics learned from the representation learning of the DRBM as well as explicit characteristics of *μ*C appearance in the first stage. Finally, we detect *μ*C clusters by aggregating the detected individual *μ*Cs with two different rules.

The key advantages of the proposed framework are as follows: 1) Improved accuracy of individual *μ*C detection based on the DRBM which automatically learns the detailed morphology of *μ*C appearances. 2) Improved efficiency of individual *μ*C detection from the fast RF classification. Not only does this stage improve efficiency, it enhances the second stage by focusing the discriminative power of the DRBM exclusively for difficult cases with subtle differences in appearance. 3) Improved accuracy of *μ*C cluster detection based on more accurate and robust individual *μ*C detection. We note that this work is an expansion of our previous work [22], which only described the DRBM classification stage.

The validity of the proposed method is evaluated and compared with relevant methods, on digitized mammograms from the original Mammographic Image Analysis Society (MIAS) database [23] and the mini-MIAS database [24], a processed version of the original, as well as digital mammograms obtained from Seoul National University Bundang Hospital (SNUBH).

## Methods

### Training The Individual Microcalcification Detector

#### Cascaded Classification Framework

**Fig 2:**
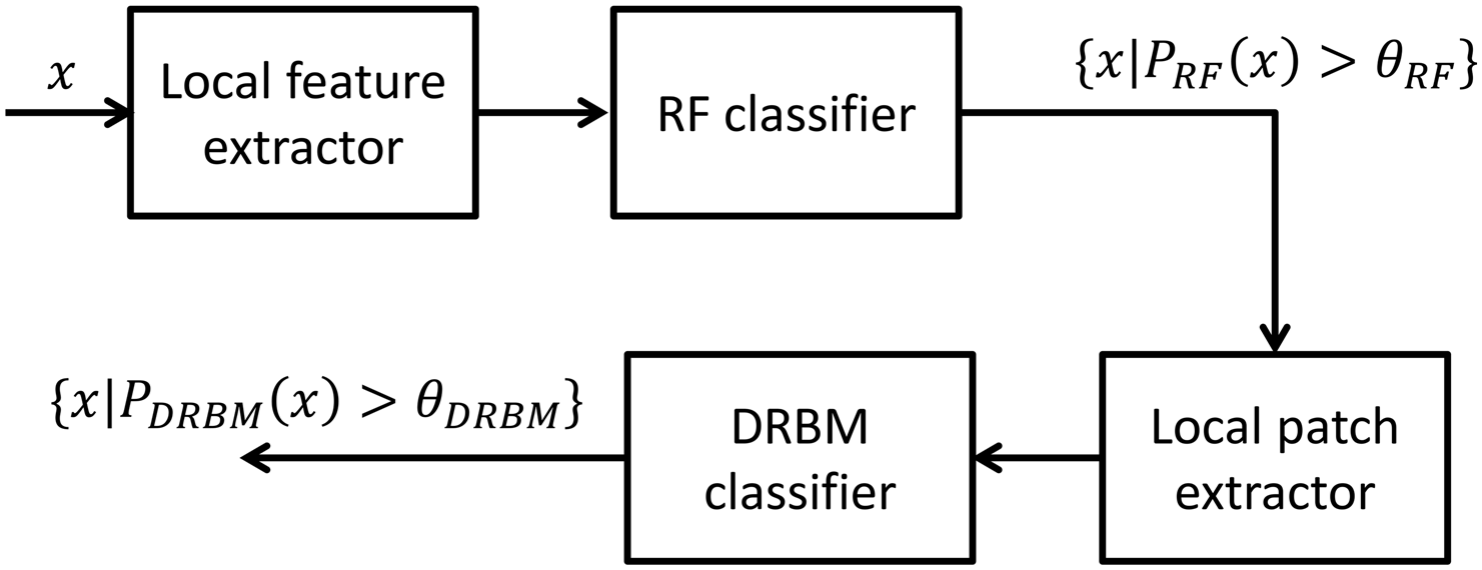
Schematic diagram of the proposed cascaded classification framework. Extracted local features from *x*, which are all pixels in a region of interest (ROI), are classified by the first RF classifier to produce *μ*C probabilities *P*_*RF*_(*x*) and *μ*C candidates are detected by thresholding these probabilities with a certain value *θ*_*RF*_. The *μ*C candidates are classified again by the second more sophisticated DRBM classifier. The input of the DRBM classifier is a local image patch and final detection results are again acquired by thresholding the probabilities *P*_*DRBM*_(*x*) with a certain value *θ*_*DRBM*_.

On a typical mammogram, only an extremely small fraction of pixels are actually *μ*Cs, as shown in Fig 1. Also, only a small number of pixels that are not *μ*Cs have similar local appearance to *μ*Cs. The appearances of most other background pixels are significantly different. Given the high resolution of recent mammography systems, it is imperative that unlikely candidates are excluded as efficiently as possible. We thus propose a two-stage cascaded classification network to speed up the whole procedure. Fig 2 shows the schematic diagram of our framework. This speed-up is achieved in the first stage by learning a highly efficient RF classifier. Even with the limited computational burden, the RF classifier has enough discriminative power to exclude most non-*μ*C pixels. The input dataset to the second stage is then small enough to be evaluated by the complex DRBM classifier. By combining the two stages, not only are we able to achieve higher efficiency, but also higher accuracy, since two classifiers cooperate to distinguish false positives.

#### Stage-1: Random Forest Classifier with Hessian Eigenvalue Features

The use of eigenvalues of the Hessian matrix as a measure of local appearance characteristics was proposed by Frangi et al. in [25]. Their focus was on tubular structures for vessel enhancement. Here, we apply a similar concept for the blob-like structure of *μ*Cs. We briefly introduce the concepts suggested in [25] and explain how we utilize it to detect *μ*Cs.

Given an image *I*, the Hessian matrix of the image computed in the neighborhood of a pixel *p* is defined as $H_{p,\sigma }=[\begin{array}{cc} \frac{\partial^{2}I\left(p,\sigma \right)}{\partial x^{2}} & \frac{\partial^{2}I\left(p,\sigma \right)}{\partial x\partial y}\\ \frac{\partial^{2}I\left(p,\sigma \right)}{\partial x\partial y} & \frac{\partial^{2}I\left(p,\sigma \right)}{\partial y^{2}} \end{array}]$. The image differentiation is calculated as a convolution with Derivatives of Gaussians (DoG) and the standard deviation of the Gaussian, *σ*, should be tuned to correspond to the size of the *μ*C to detect. By eigen-decomposition of the Hessian ***H***_*p*, *σ*_ = ***Q*** **Λ** ***Q*** ^−1^, we can compute the eigenvalues **Λ** = *diag*(λ_1_, λ_2_), (|λ_1_| < |λ_2_|) and their corresponding eigenvectors ***Q*** = (***q***_1_, ***q***_2_). The eigenvalues λ_1_ and λ_2_ represent the image gradients in the principal directions represented by the eigenvectors ***q***_1_ and ***q***_2_, and thus represent local appearance characteristics, as summarized in Table 1. We define $\lambda_{1}^{i}$ and $\lambda_{2}^{i}$ together with their ratio $R^{i}=\frac{\left|\lambda_{1}^{i}\right|}{\left|\lambda_{2}^{i}\right|}$ and the Frobenius norm of the Hessian ${\left\|H\right\|}_{F}^{i}=\sqrt{\sum_{j⩽2}\lambda_{j}^{2}}$ as the feature vector components $v^{i}=\left\{\lambda_{1}^{i},\lambda_{2}^{i},{\left\|H\right\|}_{F}^{i},R^{i}\right\}$ of the single *i*_*th*_ scale *σ*_*i*_. The reason we use the eigenvalues together with their ratio and norm is to maximize the discriminative power of the random forest. By including the norm, which represents the image gradient magnitude, and the ratio, which represents whether the local appearance is more blob-like or tubular, we can increase discriminative power and possibly reduce the depth of the decision trees in the random forest. To enable detection of *μ*Cs of multiple sizes, we construct the total feature vector by concatenating components from multiple scales as ***v*** = {***v*** ^*i*^|*i* = 1, ⋯, *n*_*σ*_}, where *n*_*σ*_ is the number of examined scales.

**Table 1:** Patterns of the value of the eigenvalues *λ*_*k*_ (H = high, L = low, N = noisy, no clear tendency and usually small, +/- indicate the sign of the eigenvalue). (|*λ*_1_| < |*λ*_2_|).

*λ*_1_	*λ*_2_	pattern
N	N	noisy
L	H-	tubular structure (bright)
L	H+	tubular structure (dark)
H-	H-	blob-like structure (bright)
H+	H+	blob-like structure (dark)

Random forest (RF) [19, 20] is an ensemble classifier comprising multiple decision trees. Each tree is learned using a randomly sampled subset *S* of the full training data set *T*. This subset *S* is inserted into the root node and recursively partitioned at each node by an optimized decision into disjoint subsets, which correspond to the left and right child nodes. The optimized decisions at each node is defined by the parameters ***θ*** = (***φ***, ***τ***), where ***φ*** is a particular dimension index among ***v*** and ***τ*** is a threshold of a binary test. Depending on the possible values of ***φ*** and ***τ***, there can be a very large number of decision candidates. The optimal decision is defined as that which maximizes the expected information gain, defined as:

$$\begin{array}{c} \theta^{opt}=\underset{\theta \in \Theta_{j}}{\arg\max}I\left(S_{j},\theta \right), \end{array}$$ (1)

where

$$\begin{array}{c} I\left(S_{j},\theta \right)=H\left(S_{j}\right)-\sum_{i\in \{L,R\}}\frac{\left|S_{j}^{i}\right|}{\left|S_{j}\right|}H\left(S_{j}^{i}\right). \end{array}$$ (2)

*S*_*j*_ denotes the subset of data samples which has reached the node *j*. *Θ*_*j*_ is the set of decision candidates for node *j*, which is often a randomly sampled subset of the all possible decisions. *L* and *R* mean left and right child nodes, respectively. $H\left(S_{j}\right)=-\sum_{c\in C}p\left(c\right)\text{log}\left(p\left(c\right)\right)$, where c is a particular class among the set of classes C, is the Shannon entropy, which represents the information gain for classification tasks. Partitioning is repeated until the subset at a particular node has homogeneous labels or its size is smaller than some predefined minimum, or if the node depth is larger than the predefined maximum. The distribution of labels of the subset at each leaf node defines the posterior class probabilities *p*(*c*|***v***) for a given feature vector ***v*** that reaches that node. The recursive splits refine the class distributions in their child nodes so that input cases are better distinguished. The forest output is computed as the average of individual trees:

$$\begin{array}{c} p\left(c|v\right)=\frac{1}{n}\sum_{i}^{n}p_{i}\left(c|v\right), \end{array}$$ (3)

where ***v*** denotes the feature vector of the test instance and *n* denotes the number of trees in our forest. We note that, in our case we have binary classes, *μ*C or non-*μ*C. We thus obtain classification by thresholding the posterior *μ*C probability. Although it should theoretically be 0.5, we can control this threshold to control the ratio of true *μ*Cs to false positives in the results.

#### Stage-2: Discriminative Restricted Boltzmann Machine Classifier

The detected candidates from the stage-1 are further classified by the DRBM classifier. Here, the raw image patch centered on the candidate pixel is used as the feature for the DRBM classifier, rather than the Hessian eigenvalue feature of the previous stage. We briefly explain the concept and model of DRBM below. We refer the reader to [21] for further details.

RBM [26] is a generative model based on a layer of hidden variables to capture a distribution over visible variables. Since RBM can effectively model common characteristics as well as discriminative features over the training data, they have been used to train features for other learning algorithms such as neural networks and SVMs. Thus, RBM is well suited for our problem of modeling the morphological ambiguity of *μ*Cs. Given a training set *D* = {(***x***_***i***_, *y*_*i*_), *i* = 1, 2, …, |*D*|} comprising the input image patches ***x***_***i***_ and their binary class labels *y*_*i*_ ∈ {0, 1}, we minimize the negative log-likelihood $L\left(D\right)=-\sum_{i=1}^{\left|D\right|}\text{log}\;p\left(y_{i,}x_{i}\right)$ to train a generative model. The joint distribution between the hidden variables ***h***, visible variables ***x***, and class label *y* is modeled as:

$$\begin{array}{c} p\left(y,x,h\right)\propto e^{-E(y,x,h)}, \end{array}$$ (4)

where

$$\begin{array}{c} E\left(y,x,h\right)=-h^{T}Wx-b^{T}x-c^{T}h-d^{T}\;\vec{y}-h^{T}U\vec{y}. \end{array}$$ (5)

**Fig 3:**
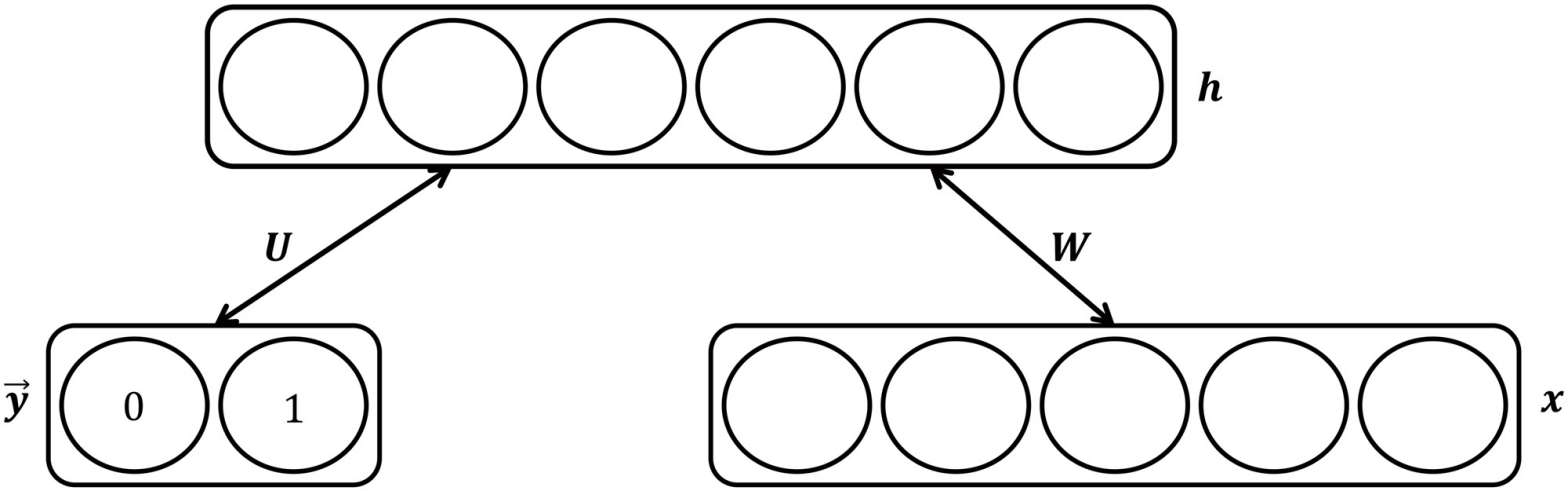
Restricted Boltzmann Machine. In training, the optimal values for weights ***W***, ***U***, which define the relations between visible variables ***x*** and hidden variables ***h***, ***h*** and the class label $\vec{y}$, respectively, and ***b***, ***c***, ***d***, which are biases in ***x***, ***h***, and $\vec{y}$, respectively, are computed. When new test data is inserted, the model computes the class label $\vec{y}$ by using the learned parameters. We note that this figure was previously presented in [21] and is reprinted here for the description of RBM.

Here, $\vec{y}$ denotes a vectorized version of the class label *y*. Specifically, given *C* possible class labels for *y*, $\vec{y}$ is a *C* dimensional vector with all zero elements except the *y*_*th*_ element, which is set to 1. The matrices ***W*** and ***U*** represent the weights representing the relation between ***x*** and ***h***, and ***x*** and $\vec{y}$, while ***b***, ***c***, and ***d*** are the vectors representing the biases for ***x***, ***h***, and $\vec{y}$, respectively. The objective of training is to optimize all the weight matrices and vectors according to *L*(*D*). The graphical model of the RBM is illustrated in Fig 3.

DRBM is a variant of the RBM made suitable for classification. For the DRBM, the conditional probability *p*(*y*|***x***) is optimized instead of the joint probability *p*(*y*, ***x***) as:

$$\begin{array}{c} L\left(D\right)=-\sum_{i=1}^{\left|D\right|}\log p(y_{i}|x_{i}). \end{array}$$ (6)

This is then used to infer the label for an input image patch. Since the DRBM is used to model features from detection candidates obtained from stage-1, the particular features that distinguish its false positives from actual *μ*Cs are learned. The *μ*C probability is obtained from the DRBM, from which we can control the final detection results by adjusting the probability threshold.

### Detecting Microcalcification Clusters

From the individual *μ*Cs detected from the cascaded classifier, we detect clusters of *μ*Cs using two different rules. The first rule, proposed by Kallergi et al. [27] and previously applied to *μ*C cluster detection by Wei et al. [15], defines a group of detected individual *μ*Cs as a true positive cluster if: 1) the distances between each pair of the *μ*Cs are less than *d*_*c*1_, and 2) three or more true *μ*Cs are located within some localized region *A*_*c*1_. If none of the *μ*Cs are true positives, then the cluster is assigned as a false positive. The second rule, proposed by Oliver et al. [13], is based on the cumulative local probability *p*_*C*_ from individual *μ*C detection probabilities *p*_*ind*_, which can be written as:

$$\begin{array}{c} p_{C}\left(x,y\right)=\int_{\Omega }p_{ind}\left(x^{\prime },y^{\prime }\right)dx^{\prime }dy^{\prime }, \end{array}$$ (7)

where *Ω* denotes again some localized region *A*_*c*2_ for which the *p*_*ind*_s are accumulated. Thresholding *p*_*C*_ with a certain value yields cluster detections. If the center of the detected cluster is inside an annotated cluster, we regard it as a true positive. In this paper, we evaluate the results based on both rules.

### Microcalcification Detection for a New Image

Given a new image, the individual *μ*Cs and their clusters are detected by the following process:

#### Breast Region Segmentation

As can be seen in Fig 1, a large portion of mammograms represent empty space. Since we are interested only in the breast region, we must first segment this region. Here, we apply a method similar to the one by Papadopoulos et al. [16], which comprises the following steps on an input image: (1) adaptive thresholding; (2) morphological dilation to smooth resulting binary labels obtained from (1); and (3) connected component analysis to detect the largest region. For the adaptive thresholding, we construct an intensity histogram and determine the threshold based on the maximum count bin *b*_*max*_. Since pixels in empty space have very similar intensities and occupy nearly half the image, they invariably correspond to *b*_*max*_. A fixed number of bins, to the right of *b*_*max*_, are examined and the threshold is determined as the intensity value that delineates the bins with the maximum declivity of the bin count. The inclusion of adaptive thresholding compared to the method of [16] helps to improve robustness since each subject has different tissue, and thus different intensity distributions.

#### Classification of Individual *μ*Cs

We first apply a learned RF classifier to all pixels in the segmented breast region in a sliding window manner. We compute Hessian matrices for each pixel, and then calculate its eigenvalues. The feature vector ***v*** is constructed from the eigenvalues for each pixel, which is then given as the input for classification by the RF.

Due to the sliding window approach, multiple positive pixel classifications can constitute a single *μ*C. Thus, the RF classification of the whole image results in a label map with multiple connected components. Since we want to obtain single coordinates corresponding to the center of each *μ*C, we perform non-maximum suppression, by determining the point that has the maximum normalized cross correlation value with a two-dimensional Gaussian kernel for each connected component.

We further classify the extracted points from the non-maximum suppression with a learned DRBM classifier. A fixed-sized raw image patch centered on the *μ*C candidate coordinate is given as the input vector for the classifier. Final detections are determined by thresholding the inferred probabilities obtained by the classifier.

#### Classification of Clusters of *μ*Cs

To detect *μ*C clusters by the rule of Kallergi et al. [27] and Wei et al. [15], for each individual *μ*C detection, we determine other *μ*C detections that are within *d*_*c*1_. If there are more than three of these *μ*Cs, we determine these *μ*Cs as a cluster. In case of overlapping clusters, we select the one with the largest number of individual *μ*C detections.

To detect clusters by the rule of Oliver et al. [13], for each pixel, we compute the cumulative local probability *p*_*C*_ of a window of fixed size, centered at that pixel, and perform thresholding to determine local *μ*C cluster windows. In case pixels with *μ*C cluster windows form a connected component, the windows form a combined *μ*C cluster where the center and the bounding box are that of the connected component.

## Results

### Mammogram Datasets

The proposed method is evaluated on three different datasets. The details of each dataset are given below.

#### SNUBH-MDB

The Seoul National University Bundang Hospital Digital Mammographic database (SNUBH-MDB) comprises 319 digital mammograms, all with spatial resolution of 0.1 mm/pixel, pixel resolution of 1914 × 2294 pixels, and 12 bit depth, from 175 clinical cases all obtained from the SNUBH using a GE Senographe 2000D digital mammography system.

Ethics approval for the study was obtained from the SNUBH for this retrospective study, and the requirement for informed consent was waived. All patient records and information were anonymized and de-identified prior to analysis.

All cases were examined by radiologists and biopsies were performed on subjects with inconclusive results. Through these clinical procedures, all mammograms were labeled as malignant, benign, or normal. For each mammogram, we achieved the bounding boxes of existing clusters of *μ*Cs from manual annotations by radiologists. For 49 randomly selected mammograms, each from different clinical cases, the pixel coordinates of individual *μ*Cs were additionally provided by the same radiologists. When training the individual *μ*C detector, positive data were sampled from coordinates of individual *μ*C, while negative data were sampled randomly from breast regions excluding the bounding box of *μ*C clusters. For brevity, we refer to these 49 mammograms as SNUBH-MDB-*μ*Ci and the remaining 270 mammograms, from 126 clinical cases, as SNUBH-MDB-*μ*Cc from this point on. The SNUBH-MDB-*μ*Ci is composed of 20 benign (from 20 clinical cases) and 29 malignant (from 29 clinical cases) mammograms. The SNUBH-MDB-*μ*Cc is composed of 3 types of data, specifically, 106 benign (from 54 clinical cases), 62 malignant (from 32 clinical cases), and 102 normal (from 40 clinical cases) mammograms. While biopsies were not performed on normal cases, they can be further divided into cases with and without *μ*Cs. *μ*Cs in normal cases have unclear boundaries and are hard to detect. Overall, the SNUBH-MDB-*μ*Cc contains more challenging and wide range of patients than the SNUBH-MDB-*μ*Ci.

#### MIAS DB

The Mammographic Image Analysis Society (MIAS) database [23] comprises 207 normal mammograms, 90 mammograms containing abnormalities including masses, spiculated masses, and architectural distortions, but without *μ*Cs, and 25 mammograms containing a total of 28 clusters of *μ*Cs. The total 322 images are of spatial resolution 0.05 mm/pixel and optical density linear in the range 0 – 3.2 and quantised to 8 bits. The size of the mammograms were either small (1600 × 4320 pixels), medium (2048 × 4320 pixels), large (2600 × 4320 pixels), or extra-large (5200 × 4320 pixels). For images containing *μ*Cs, the center coordinate and the radius of the circle enclosing the *μ*C cluster are included in the original database. To train our individual *μ*C detector, we used the annotations of the individual *μ*Cs used in [13], provided by its authors.

#### Mini-MIAS DB

The mini-MIAS database [24] is a reduced, clipped and padded version of the original, where the spatial resolution is reduced to 0.2 mm/pixel, and the sizes of all images are fixed to 1024 × 1024 pixels. While it includes the same 322 images, *μ*C cluster annotations are provided for only 23 mammograms. We note that due to the differences in image configuration, we obtained annotations of individual *μ*Cs (as pixel coordinates) from radiologists, independently from that we had obtained for the original MIAS DB.

#### Access to DBs

Mammograms and all annotations of the SNUBH-MDB-*μ*Ci, mammograms of the MIAS DB, and individual *μ*C annotations of the mini-MIAS DB are available at http://dx.doi.org/10.5061/dryad.jm6k3. Mammograms and annotations of SNUBH-MDB-*μ*Cc are available upon request for researchers who meet the criteria for access to confidential data. Requests should be made to Sun Mi Kim (kimsmlms@daum.net), see http://cv.snu.ac.kr/research/cascade-mc-detector15/index.html for more information. Mammograms and *μ*C cluster annotations of the mini-MIAS DB are available at http://peipa.essex.ac.uk/info/mias.html. Individual *μ*C annotations of the MIAS DB are available at http://eia.udg.edu/~aoliver/mammoWeb/lesMic.html.

### Experimental Settings

#### Training Sets

A positive and negative set of feature vectors of individual *μ*Cs are required for training the individual *μ*Cs classifier. Since we have obtained expert annotations for the SNUBH-MDB, MIAS DB, and the mini-MIAS DB, it is straightforward to construct the positive sets, which comprises the features at the annotated *μ*C coordinates. This same set is applied for both the stage-1 RF and stage-2 DRBM classifiers.

To construct the negative set of the first stage RF classifier, we randomly sample coordinates from the whole breast region except the bounding boxes of *μ*C cluster, for the SNUBH-MDB and the mini-MIAS DB. For the MIAS DB, the obtained expert annotations also include coordinates of negative samples. Thus we use these coordinates to construct the negative set in this case.

For the negative set of the stage-2 DRBM classifier, we sample from the false positives of the stage-1 RF classifier. This is the same for all datasets. We set the size of the negative set to be the same as that of the stage-1 RF classifier.

#### Parameters

Parameter values are empirically set for each different dataset. The set of scales in stage-1 RF are fixed as *σ* = {1}, *σ* = {1, 2, 3, 4, 5}, *σ* = {0.5, 1, 1.5, 2, 2.5}, for the SNUBH-MDB, MIAS DB, and the mini-MIAS DB, respectively. The RF in the stage-1 RF, comprises 100 trees, each with maximum depth 30. For each tree, the training is stopped when the maximum depth is reached or when no node can be split without resulting in a child with corresponding dataset size smaller than the 1% of the training set size. The probability threshold of the RF classifier, which can be adjusted to optimize the stage-2 DRBM classification performance, is also empirically determined as 0.3, 0.5, and 0.5 for the SNUBH-MDB, MIAS DB, and the mini-MIAS DB, respectively. We note that, the threshold value 0.3 for the SNUBH-MDB is smaller than 0.5, which means that the *μ*C probability $p_{\mu C}^{RF}$ can become smaller than the non-*μ*C probability $p_{{\mu C}^{c}}^{RF}=1-p_{\mu C}^{RF}$. This is because the emphasis in this stage is on minimising false negatives, even if some non-*μ*Cs incorrectly pass, since they can be excluded in the next stage.

For the stage-2 DRBM classifier, which is trained by contrastive divergence [28], we have to tune many hyper-parameters including the size of the visible layer, the size of the hidden layer, the learning rate, and the number of iterations over the training set [21]. The optimal values for the hyper-parameters were determined by a grid-like search on a validation set. The values of visible layer size (input patch size), hidden layer size, learning rate, and the number of iterations, used for evaluation were, 225 (15 × 15), 50, 0.02, 100 for the SNUBH-MDB, 841 (29 × 29), 200, 0.02, 30 for the MIAS DB, and 81 (9 × 9), 200, 0.04, 30 for the mini-MIAS DB, respectively. When training, we use “mini-batches” for faster convergence. For more details regarding this process, we refer the reader to [21] and [29].

For *μ*C cluster detection, we set as *d*_*c*1_ = 0.4*cm* and *A*_*c*1_ = 1*cm* ^2^ for the first rule of Wei et al. [15] and *A*_*c*2_ = 1*cm* ^2^ for the second rule of Oliver et al. [13], all of which were the values used in the respective original works. In terms of the mammograms used in our experimental evaluation, the area of 1*cm* ^2^ corresponds to 100 × 100, 200 × 200, and 50 × 50 pixels for the SNUBH-MDB, MIAS DB, and mini-MIAS DB, respectively.

### Quantitative Evaluation

We evaluate the accuracy of 1) the individual *μ*C detection step and 2) the *μ*C cluster detection framework. Performance of our method is compared to the methods of Wei et al. [15] and Oliver et al. [13]. For both methods, we use our own implementations. We have carefully followed the descriptions in the papers during implementation, and adapt the parameters to each database for the best results.

#### Evaluation of individual *μ*C detection framework

Since annotations of individual *μ*Cs must be given and the images containing *μ*Cs are limited, especially for the MIAS DB and mini-MIAS DB, we only evaluate individual *μ*C detection accuracy on the SNUBH-MDB-*μ*Ci, by 10-fold cross validation. In cross validation, the whole dataset is divided into 10 different groups, where one group is designated for the test, and the remaining groups are used to train the classifier. This process is repeated 10 times so that testing is performed at least once for each group.

Quantitative performance is measured by constructing the receiver operating characteristic (ROC) curve as well as the precision-recall curve. The ROC curve is a plot of sensitivity, i.e., the ratio of *μ*Cs actually detected among all *μ*Cs present in the images, against (1-specificity), i.e., the ratio of false positive detections among all non-*μ*C pixels. Precision-recall curve is a plot of precision, i.e., the ratio of true *μ*Cs among all positive detections, against recall, which is identical to sensitivity. Different values of sensitivity/specificity and precision/recall are obtained at different operating points. Here, the operating point is controlled by a single parameter, namely, the threshold for the probability inferred by the stage-2 DRBM classifier.

**Fig 4:**
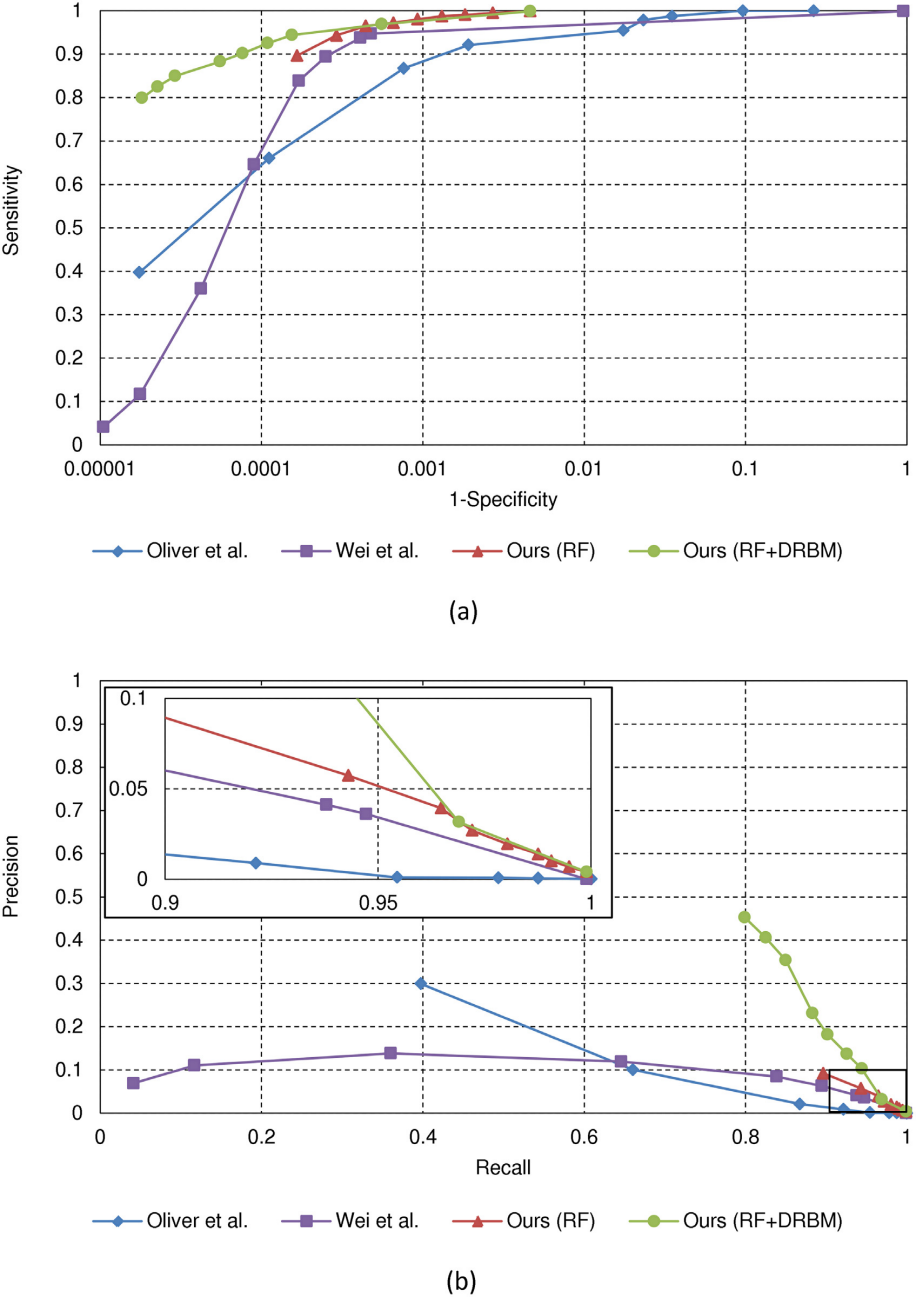
Quantitative results on the individual *μ*C detection in the SNUBH-MDB-*μ*Ci. (a) ROC curve. The x-axis (FP rate) is represented on a log-scale. (b) Precision-Recall curve.

Fig 4 shows the ROC and precision-recall curves for the methods in [13, 15], the stage-1 RF classifier, and the proposed cascaded classifier. For both ROC and precision-recall, a higher curve represents better performance. We can easily see that our first stage RF classifier has slightly higher accuracy than the methods of [13] and [15], while the proposed cascaded classifier clearly performs better than the previous methods. In terms of the ROC curve, with the same sensitivity value of 0.9 as the methods of [13, 15], the stage-1 RF classifier, and the proposed cascaded classifier, the (1-specificity) values are 0.00146, 0.00027, 0.00018, and 0.00007, respectively. This means that when the number of true positives is 869 among a total of 966 *μ*Cs, the number of false positives are 75036, 13902, 9025, and 3819, respectively. In terms of the precision-recall curve, precisions are 0.01, 0.06, 0.09, and 0.19 for a same recall value of 0.9 for the methods of [13, 15], the stage-1 RF classifier, and the proposed cascaded classifier, respectively. This means that to correctly detect 869 true positives among all target *μ*Cs, the total numbers of detections obtained are 63414, 14435, 9719, and 4665. This also means that the proposed cascaded classifier only gives 6% and 28% of false positives compared to [13] and [15], respectively, when detecting a similar rate of *μ*Cs.

#### Evaluation of *μ*C cluster detection framework

The detection accuracy is evaluated by comparing the free-response receiver operating characteristic (FROC) curves [30] of the different methods. The FROC curve plots the sensitivity on the cluster detection, i.e., the ratio of *μ*C clusters actually detected among all *μ*C clusters present in the images, against the average number of false positive clusters per image. A higher FROC curve represents better performance.

We evaluate the detection accuracy based on the first rule, from [15, 27], on the SNUBH-MDB-*μ*Ci, by 10-fold cross validation. For this rule, the operating point is controlled by thresholding the probability for individual *μ*C detection. Using the second rule from [13], the evaluations are performed on the SNUBH-MDB-*μ*Ci, SNUBH-MDB-*μ*Cc, MIAS DB, and mini-MIAS DB. Again, 10-fold cross validations are performed for SNUBH-MDB-*μ*Ci, MIAS DB, and mini-MIAS DB. We note that, for the MIAS DB and mini-MIAS DB, images with and without *μ*Cs are each divided into 10 groups of uniform size, so that testing is performed on images without *μ*Cs as well. For the SNUBH-MDB-*μ*Cc, training is performed on the SNUBH-MDB-*μ*Ci, thus constituting a more realistic experimental setup. For the second rule, the operating point is controlled by the threshold for the cumulative local probability *p*_*C*_.

**Fig 5:**
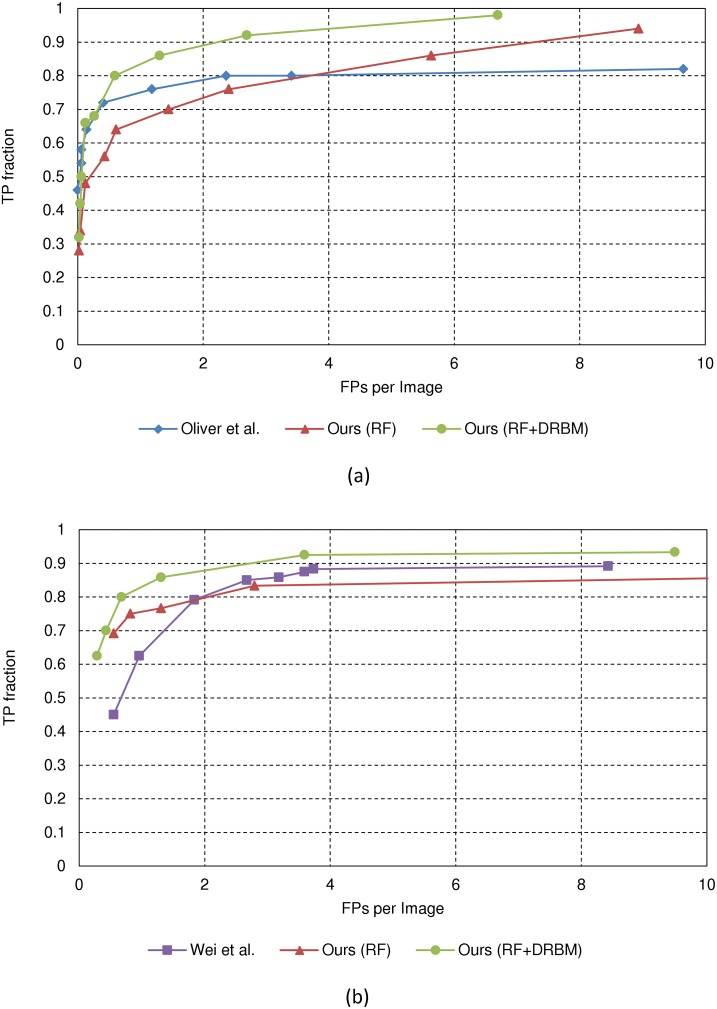
FROC curves obtained by 10-fold cross-validation on the SNUBH-MDB-*μ*Ci. Two representative rules for the cluster detection are utilized here. (a) FROC curves by the rules used in [13]. (b) FROC curves by the ruled used in [15].

Fig 5 shows the FROC curves for the SNUBH-MDB-*μ*Ci. In Fig 5(a), the cluster detection results based on the individual detection results of the stage-1 RF classifier and the cascade classifier, using the same rule as in [13], are compared with the method of [13]. The AUC values of each method are 0.82, 0.92, and 0.79. In Fig 5(b), the results using the same rule as in [15] are compared with the method of [15]. The AUC values of each method are 0.82, 0.91, and 0.84. Overall, the cluster detection results based on the stage-1 RF classifier are comparable to the methods of [13] and [15]. On the other hand, the results based on the cascade classifier clearly outperform the previous methods. The fraction of true positives is 3%—20% higher than the method of [13], and 3%—25% higher than the method of [15], for a similar number of false positives.

**Fig 6:**
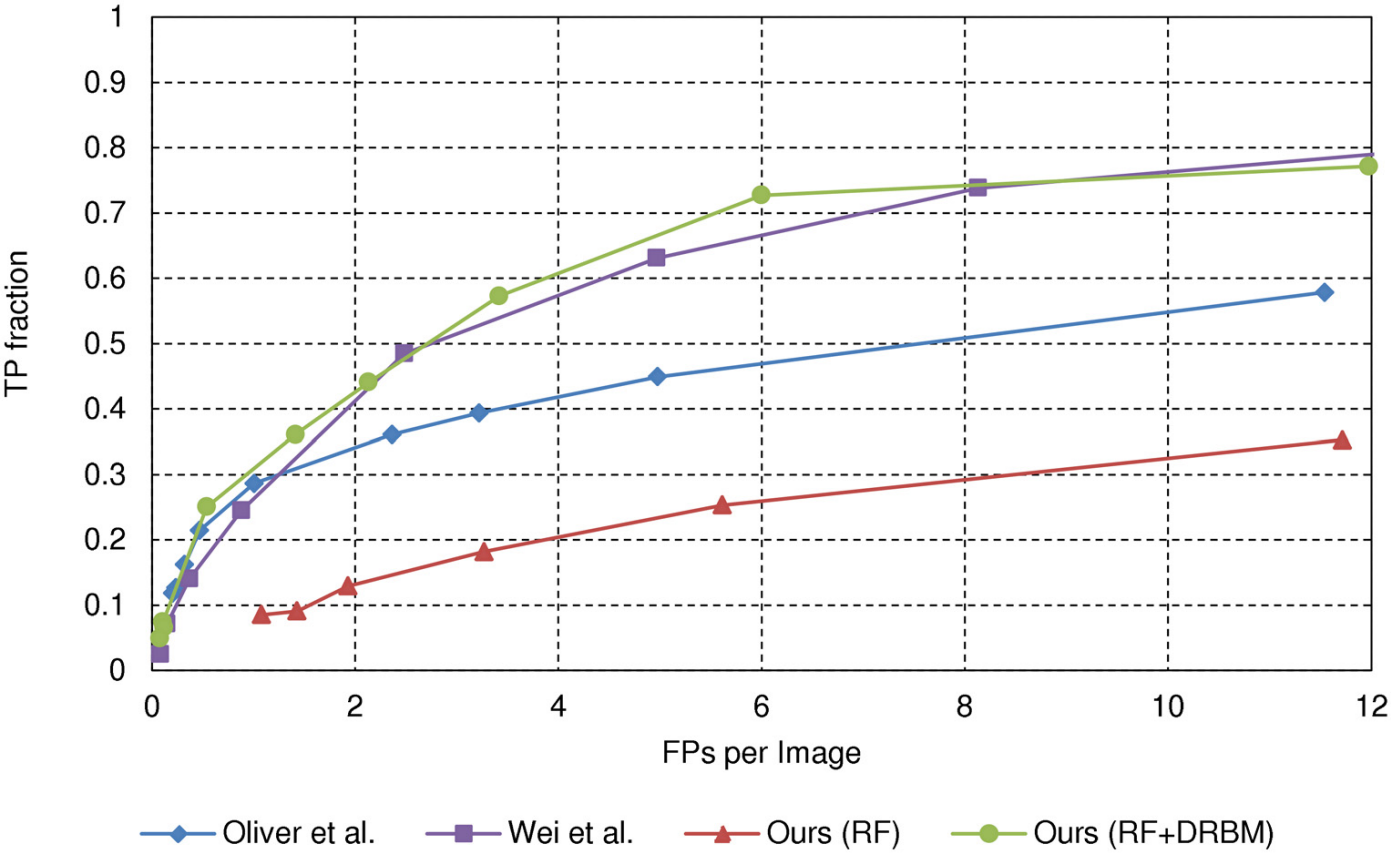
FROC curves for the SNUBH-MDB-*μ*Cc. The SNUBH-MDB-*μ*Cc can be evaluated only by the rule in [13] since we have only cluster annotations for the SNUBH-MDB-*μ*Cc.

Fig 6 shows the FROC curves for the SNUBH-MDB-*μ*Cc, using the rule in [13]. The rule in [15] is inapplicable since there are no annotations of individual *μ*Cs for this dataset. Here, we can see that although the stage-1 RF classifier results are much worse, the cascade classifier results are much better than that of [13], and slightly better than [15], with the AUC value 0.63 for the proposed method and 0.60 for [15], respectively.

We note that the substantial performance decrease of all methods for the SNUBH-MDB-*μ*Cc compared to that for the SNUBH-MDB-*μ*Ci has two main causes. First, the SNUBH-MDB-*μ*Cc also contains normal cases, which are not included in the SNUBH-MDB-*μ*Ci. Since these cases were not included in the training, the trained classifier may be insufficient to handle these cases. Second, while the methods were trained with the SNUBH-MDB-*μ*Ci, which only contains craniocaudal (CC) view images, the SNUBH-MDB-*μ*Cc also contains mediolateral oblique (MLO) view images. Since positive and negative training samples are only collected from the CC view images, the trained classifier may be inadequate for local appearances exclusive to MLO view images.

**Fig 7:**
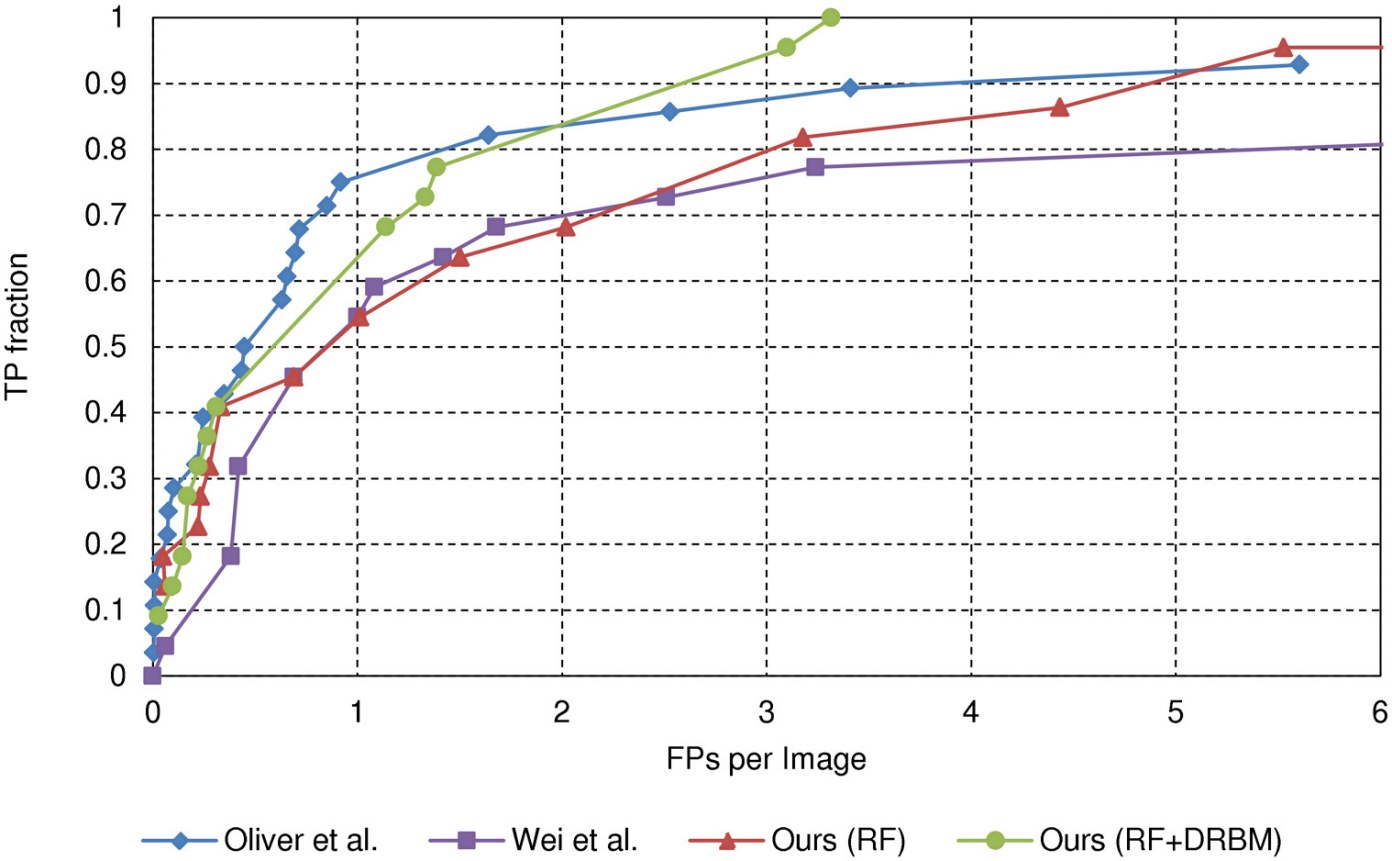
FROC curves for the MIAS database. All 322 images including mammograms with *μ*Cs and without *μ*Cs are evaluated by the rule in [13].

Fig 7 shows the FROC curves for the MIAS DB. It shows that the proposed cascade classifier and the method of [13] show comparable accuracy. These methods clearly outperform the stage-1 RF classifier and the method of [15]. To analyse the significance of the results, we compute 95% confidence intervals, following the parametric procedure of [31]. At sensitivity 0.8, the false positives are in the range of [1.04, 2.88] by the proposed cascade method and [0.96, 1.73] for the method of [13]. At sensitivity 0.9, the ranges are [1.4, 3.19] and [3.23, 5.52], respectively. We can see that the proposed method outperforms the method of [13] for higher sensitivity values. The proposed cascade classifier is able to return all true *μ*C clusters, if more than 3.3 false positives per image are allowed on average. Overall, the proposed method performs slightly better, in terms of area under the curve (AUC) value, with 0.84, compared to [13], with 0.81. We note that, for this dataset, the results of the method [13], provided by the authors, are from their original implementation.

**Fig 8:**
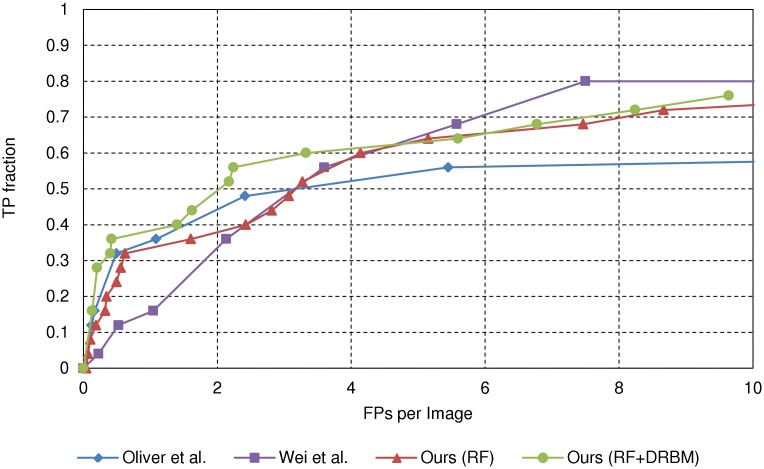
FROC curves for the mini-MIAS database. All 322 images including mammograms with *μ*Cs and without *μ*Cs are evaluated by the rule in [13].

In comparison, we observe that the FROC curves in Fig 8 are considerably lower than those from the MIAS DB in Fig 7. As previously mentioned, due to the reduced resolution, the *μ*Cs in the mini-MIAS DB have considerably smaller pixel area compared to the original MIAS DB. This diminishes spatial cues required to distinguish *μ*Cs from noise or other tissue with similar appearance. This may result in limited accuracy for low resolution images. Thus, the proposed method requires high resolution images for optimal performance. Although the proposed cascade classifier and the method of [15] show comparable accuracy, the proposed method performs better at operating points with less than four false positives per image. Overall, the proposed method, with AUC 0.62, performs better than the method of [15], with AUC 0.57.

### Qualitative Evaluation

#### Evaluation of individual *μ*C detection framework

**Fig 9:**
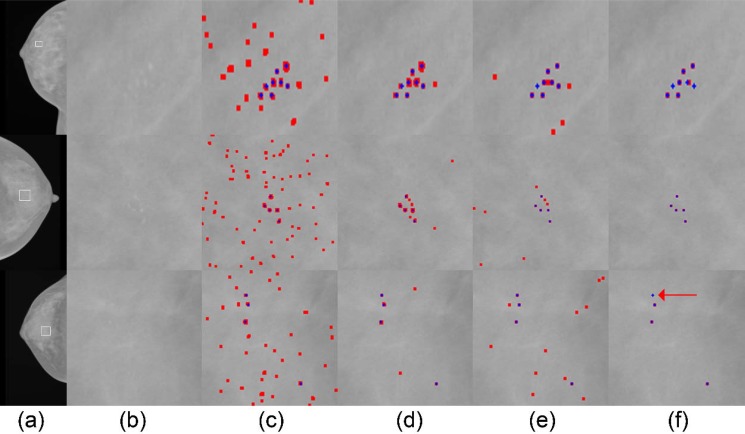
Qualitative results on the individual *μ*C detection in the SNUBH-MDB-*μ*Ci. Each row represents a different case and each column shows (a) Test images, (b) Expanded views of ROIs (bounded by white boxes in (a)) containing annotated *μ*Cs and corresponding detection results by the methods of (c) Oliver et al. [13], (d) Wei et al. [15], (e) Ours (RF), (f) Ours (RF+DRBM). Blue crosses are annotated individual *μ*Cs and red pixels in the detection results are detected points by the methods. Best viewed in color.

Fig 9 shows individual *μ*C detection results for three example cases from the SNUBH-MDB-*μ*Ci in each row. The whole and zoomed views of the images are shown in Fig 9(a) and 9(b), respectively. We can see that the *μ*Cs are difficult to localize due to its unclear boundaries. Compared to the results of the stage-1 RF classifier, shown in Fig 9(e), the numbers of false positives are greatly reduced in the cascade classifier, shown in Fig 9(f). This shows the effectiveness of the higher level DRBM classifier. For these particular cases, the method of [13] detects more false positives compared to the method of [15], but both methods detect higher number false positives compared to the proposed cascade classifier. However, there is still room for improvement. In the case shown in the bottom row, the cascade classifier fails to detect a true *μ*C, highlighted by an arrow, which results in a *μ*C cluster detection failure.

#### Evaluation of *μ*C cluster detection framework

**Fig 10:**
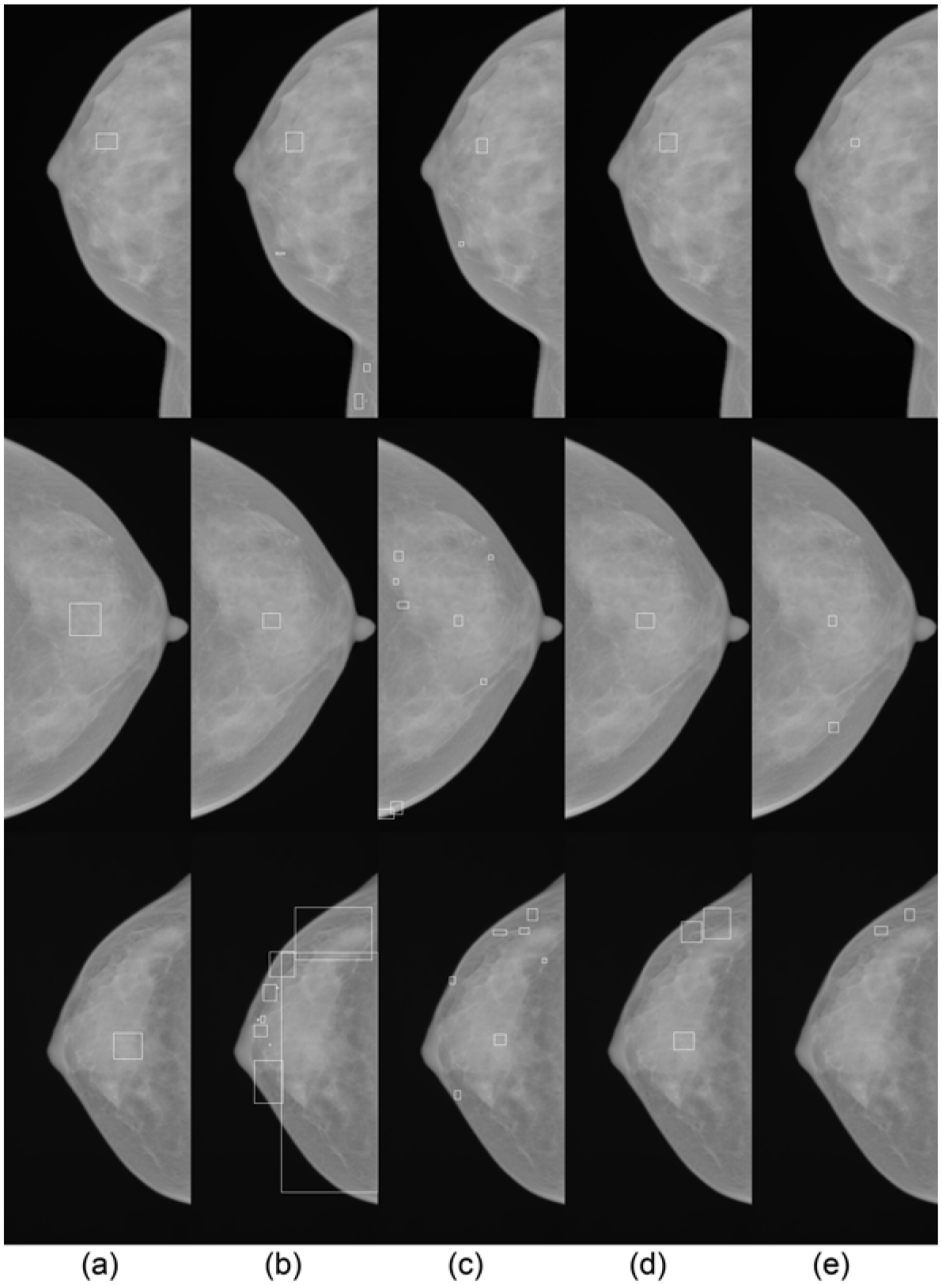
Qualitative results on the *μ*C cluster detection in the SNUBH-MDB-*μ*Ci. Each row represents a different case which corresponds to the case in the same row in Fig 9 and each column shows (a) annotated clusters and cluster detection results by the methods of (b) Oliver et al. [13], (c) Wei et al. [15], and (d) Ours (RF+DRBM) with the criterion of Oliver et al. [13], and (e) Ours (RF+DRBM) with the criterion of Wei et al. [15].

Fig 10 shows *μ*C cluster detection results for the same cases as in Fig 9. Here, the cluster detection rule of [13] is used for the method of [13], while the rule of [15] is used for the method of [15]. For the proposed cascade classifier, results using both rules are shown. For the top and middle row cases, all methods correctly detect the *μ*C cluster, but the proposed method with the rule of [13] detects the smallest number of false positives. For the bottom row case, all methods detect the correct *μ*C cluster, except the proposed, using the rule of [15]. The missed detection is caused by the aforementioned missed individual *μ*C detection. Again, comparing the number of false positives and bounding box sizes, the proposed method with the rule of [13] shows the best results.

**Fig 11:**
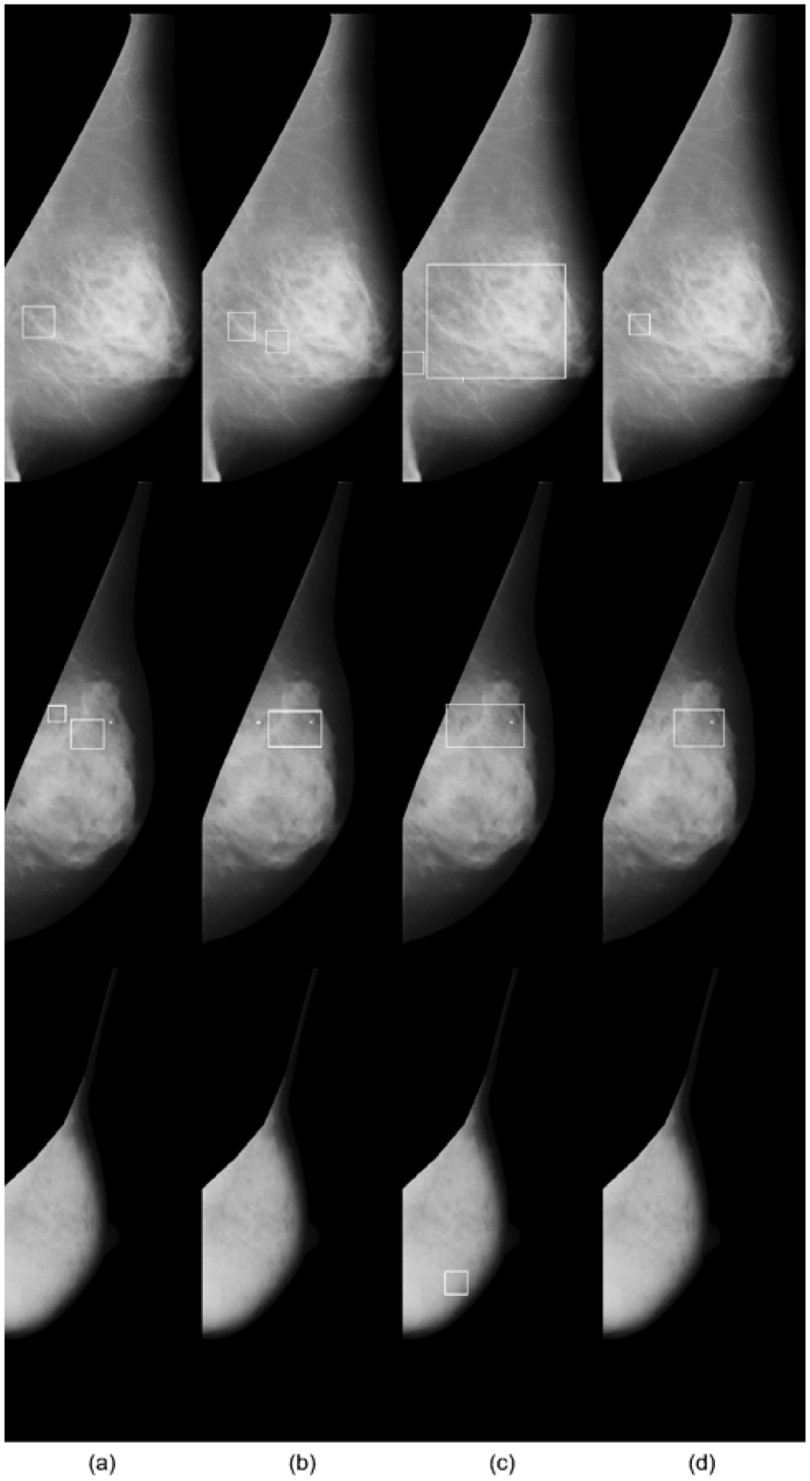
Qualitative results on the *μ*C cluster detection in the MIAS database. Each row represents a different case and each column shows (a) annotated clusters and cluster detection results by the methods of (b) Oliver et al. [13], (c) Wei et al. [15], and (d) Ours (RF+DRBM). The cluster detections of all methods are conducted by the rule in [13]. For the bottom row case, there are no *μ*C clusters.

Fig 11 shows *μ*C cluster detection results for three example cases from the MIAS DB in each row. Comparing the bounding boxes and number of false positives, we can see that the proposed cascaded classifier gives the best results for the top and bottom row cases. For the middle row case, all three methods are comparable. While quantitative results of the method [13] were from the implementation original authors, these results are from our own implementation.

**Fig 12:**
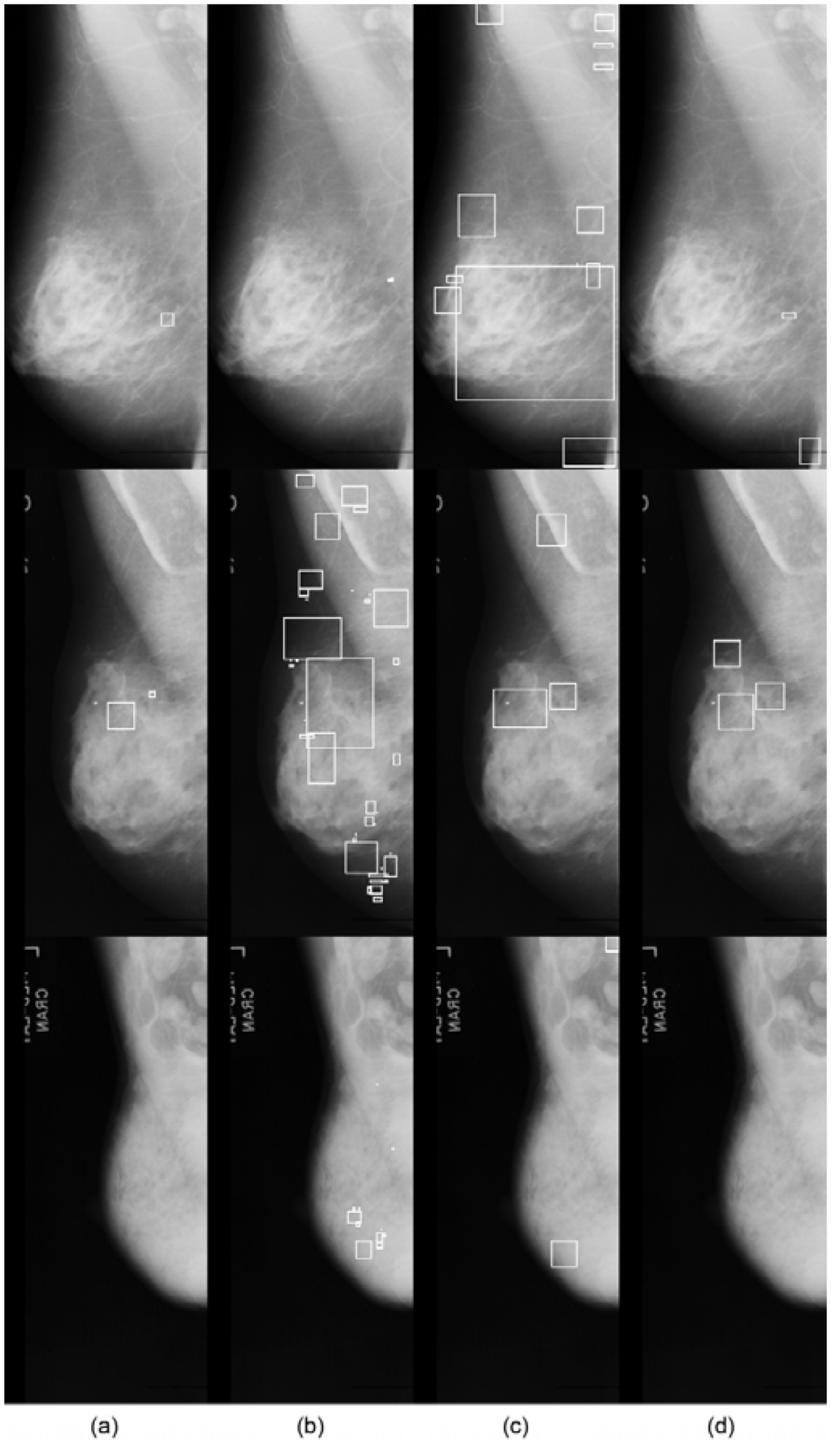
Qualitative results on the *μ*C cluster detection in the mini-MIAS database. Each row represents a different case which is a processed version of the case in the same row in Fig 11 and each column shows (a) annotated clusters and cluster detection results by the methods of (b) Oliver et al. [13], (c) Wei et al. [15], and (d) Ours (RF+DRBM). The cluster detections of all methods are conducted by the rule in [13] same as in the MIAS database.

Also, Fig 12 shows *μ*C cluster detection results for the same example cases, but from using the images of the mini-MIAS DB, shown in Fig 11. We can see that the proposed cascaded classifier gives the best results for all three cases. The performance degradation compared to the results of the MIAS DB can also be observed.

### Computational Complexity

The computational complexity at test time of the proposed stage-1 RF classifier is linearly proportional to the number of pixels as each pixel is classified independently. Most of the computational burden is in computing the hessian matrix, where Gaussian convolution is performed for numerical stability, as in [25]. This convolution depends on the number of scales and the *σ* values. Once the hessian is computed, the eigenvalues can be computed by two equations. Also, the prediction using the random forest requires a small number of boolean operations for each pixel, which is the maximum tree depth, 30, multiplied by the number of trees, 100.

The stage-2 DRBM classifier generally requires more computation for a single input than the previous stage, though it depends on the input patch size, the number of hidden nodes, etc. However, since the number of predictions is only a fraction (less than 1%, on average) of the total number of pixels, this stage requires significantly less computation than the previous stage.

While stage-1 RF and stage-2 DRBM classifiers are both quite efficient at test time, training does require much more computation. However, this can be performed offline, and does not affect the inference at test time.

Table 2 shows the average computation time, in seconds, for detection of individual *μ*Cs from images of the SNUBH-MDB, for the methods of [13, 15] and the proposed cascade method. All the methods are implemented in C++ and tested on a 3.3 GHz CPU with 16GB RAM and no GPU support. The processing time for *μ*C cluster detection is excluded since it is identical for all methods. We note that our proposed method is 55× faster than the method of [13] and is comparable to that of [15] while outperforming both methods in terms of accuracy. Within the proposed method, the stage-2 DRBM classifier constitutes less than 3% of the total time.

**Table 2:** The computation times of our and baseline algorithms. The Test time is the average time for detecting *μ*Cs in one image. The time is represented in seconds.

	Oliver et al.	Wei et al.	Ours (RF)	Ours (RF+DRBM)
Test time (s)	2020.86	35.92	35.05	36.03

## Discussion

We have shown that the proposed cascade classifier outperform previous methods [13, 15], in terms of accuracy, for the SNUBH-MDB and the MIAS DB. However, this is less evident for the mini-MIAS DB, which has a much lower spatial resolution, and thus, gives lower detection accuracy for all three methods. Based on these results, we observe that not only does our method perform better, it has a higher comparative advantage, for mammograms with higher spatial resolution. The performance degradation of the proposed method at lower resolutions is due to the instabilities of Hessian eigenvalues along with the difficulties in capturing subtle differences of structures with smaller pixel dimensions. Thus, mammograms with high spatial resolution should be achieved to maximize the performance of the proposed method.

For future works, the stage-1 RF classifier can be significantly accelerated by utilizing a GPU, as described in [32]. Also, we hope to utilize different methods [33–35] for training the classifier. The proposed method can also be extended for tomosynthesis.
